# Pigmented Villonodular Synovitis Causing Osteonecrosis of the Femoral Head: A Case Report

**DOI:** 10.1155/2013/756954

**Published:** 2013-12-12

**Authors:** Tomohiro Mimura, Taku Kawasaki, Keitaro Yagi, Kanji Mori, Shinji Imai, Yoshitaka Matsusue

**Affiliations:** Department of Orthopaedic Surgery, Shiga University of Medical Science, Tsukinowa-cho, Seta, Otsu, Shiga 520-2192, Japan

## Abstract

We report a case of a 27-year-old man with pigmented villonodular synovitis of the hip joint with coincident osteonecrosis of the femoral head. According to our review of the English-language literature, no detailed report of osteonecrosis of the femoral head complicated with pigmented villonodular synovitis has been published. Preoperative X-ray images showed joint narrowing and severe multiple bone erosions at the acetabulum and femoral neck. Magnetic resonance imaging revealed a low-intensity band attributable to osteonecrosis of the femoral head and massive diffuse pigmented villonodular synovitis lesions. Comparison of a three-dimensional computed tomographic image of this patient with an angiographic image of a normal individual demonstrated proximity of the pigmented villonodular synovitis-induced bone erosions to the medial and lateral femoral circumflex arteries and retinacular arteries, suggesting likely the compromise of the latter by the former. We propose that the massive pigmented villonodular synovitis may have contributed to the pathogenesis of osteonecrosis of the femoral head in this patient. We performed open synovectomy and total hip arthroplasty. No operative complications occurred, and no recurrence of the pigmented villonodular synovitis was detected for 3 years after the operation.

## 1. Introduction

Pigmented villonodular synovitis (PVNS) was named and described by Jaffe et al. in 1941 [1]. However, the first description of PVNS is commonly attributed to Chassaignac in 1852. Myers and Masi estimated that the overall prevalence of PVNS is 1.8 per million people [2]. They reported that the knee is the most commonly affected joint, accounting for up to 80% of cases, and that the hip is the second most commonly affected joint, accounting for 15%. In contrast, osteonecrosis of the femoral head (ONFH) is a relatively common disease, first described by Heimann and Freiberger in 1960 [3]. We herein report a case of ONFH complicated with PVNS, which, to our knowledge, is the first detailed report of this presentation in the English-language literature. We propose that the massive PVNS may have contributed to the occurrence of ONFH in this patient, creating multiple bone erosions around the femoral neck.

## 2. Case Presentation

**Figure 1:**
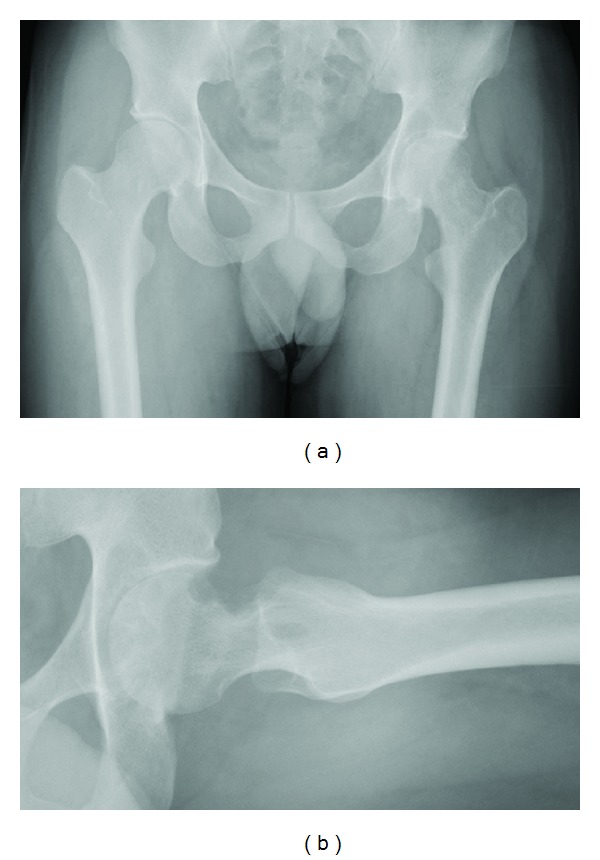
(a, b) Joint narrowing and multiple bone erosions of the hip joint are seen on this roentgenogram.

**Figure 2:**
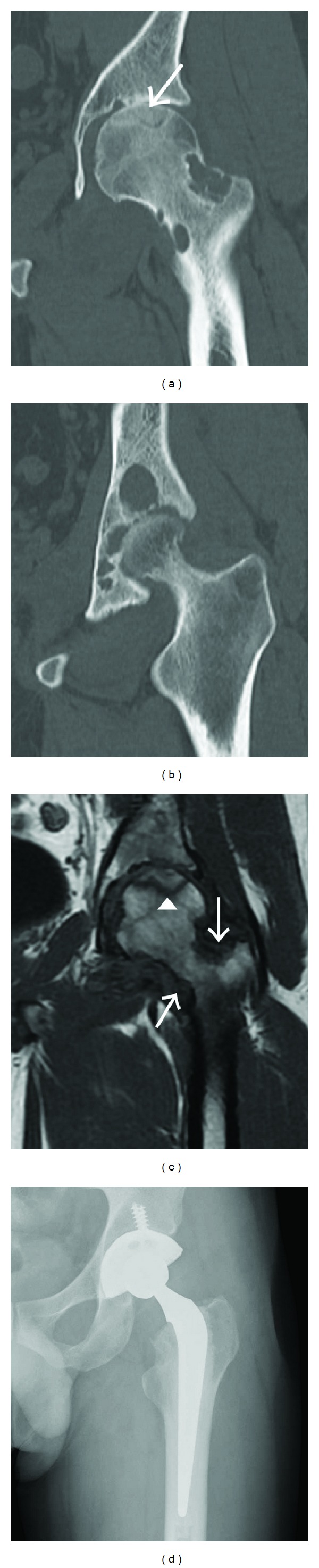
(a, b) Computed tomography image showing the femoral neck and acetabular bone erosions, and area of bone necrosis (arrow). (c) Magnetic resonance image showing a typical low-intensity band of osteonecrosis of the femoral head (arrowhead) and mass lesions (arrows) around the femoral neck on T1-weighted imaging. (d) The patient underwent total hip arthroplasty with complete synovectomy and tumor resection using an argon laser.

The patient was a 27-year-old man (weight: 66 kg; height; 166 cm) with no underlying disease. This patient became aware of coxalgia 1 year earlier. The coxalgia was associated with continuous pain that became more severe, and at the time of presentation he could not walk without an axillary crutch. X-ray images showed joint narrowing and multiple bone erosions of the left hip joint (Figure 1). Computed tomography (CT) clearly showed femoral neck and acetabular bone erosions as well as ONFH (Figures 2(a) and 2(b)). In contrast, magnetic resonance (MR) imaging showed ONFH and mass lesions around the femoral neck. T2-weighted fat-suppressed MR imaging also revealed an irregular tumoral lesion (Figure 2(c)). The patient had type C ONFH according to the Nishii et al. classification [4] and stage 4 ONFH according to the Association Research Circulation Osseous classification [5].

We performed an arthroscopic biopsy before radical treatment. Histological analysis of the synovial tissue showed diffuse PVNS. The patient subsequently underwent total hip arthroplasty (THA) (KYOCERA Medical Corporation, Osaka, Japan) with complete synovectomy and tumor resection using an argon laser (Figure 2(d)). Severe invasive PVNS was seen in the inferior space of the hip joint, particularly around the femoral neck.

Pathological findings from an analysis of permanent preparations of the synovium and femoral head were consistent with PVNS and avascular bone necrosis, respectively. Proliferative histiocyte-like mononuclear cells were observed with multinucleated giant cells in the fibrotic stroma. Hemosiderin-laden macrophages and foamy cells were also occasionally observed. The subchondral zone of the femoral head showed avascular bone necrosis. Both empty lacunae and fat necrosis were found in the resected femoral head. Fibrosis and proliferation of vessels were observed around the necrotic trabeculae, and new bone apposition was present.

After the procedure, the Harris hip score improved from 79 to 97. No operative complications occurred, and no recurrence of PVNS was detected for 3 years after the procedure.

## 3. Discussion

PVNS is a rare disease and is typically present in young adults between 20 and 40 years of age. PVNS usually affects one joint; involvement of more than one joint is extremely rare [6]. In their paper, Cotton et al. reported that both hips were affected in only 2 of 58 patients [7]. Several previous studies have reported that PVNS is slightly more prevalent in female patients [8]. In the hip joint, the diffuse type is more common than the local type [9]. The predominant roentgenogram findings of PVNS in the hip joint are multiple cyst-like lesions or erosive areas of non-weight-bearing regions of the acetabulum and femoral head and/or femoral neck. Cotton et al. showed the presence of these bony lesions in 95% of their cases (cortical erosion, 43%; cyst-like erosion, 90%) [7].

The etiology of PVNS is unclear. Many theories have been put forward to explain the pathogenesis of PVNS, including etiopathological mechanisms involving inflammatory reactions [1], abnormal cellular and humoral immunity, genetic predisposition, or recurrent hemorrhage following trauma. However, a neoplastic origin currently seems to be the most influential theory [10]. Several reports have described the development of local recurrence and metastasis of PVNS [11]. At present, adjuvant radiation therapy is used to treat aggressive PVNS [11]. However, complete synovectomy and arthroplasty have been used as alternative treatments of PVNS. In previous studies, outcomes have been better after synovectomy with THA than after synovectomy alone [12, 13]. Yoo et al. reported no recurrent PVNS in their eight cases treated with cementless THA during an average followup of 8.9 years [14]. They concluded that THA with synovectomy is an adequate therapeutic choice for patients with PVNS demonstrating end-stage joint destruction and appears to effectively improve clinical results and prevent disease recurrence.

**Figure 3:**
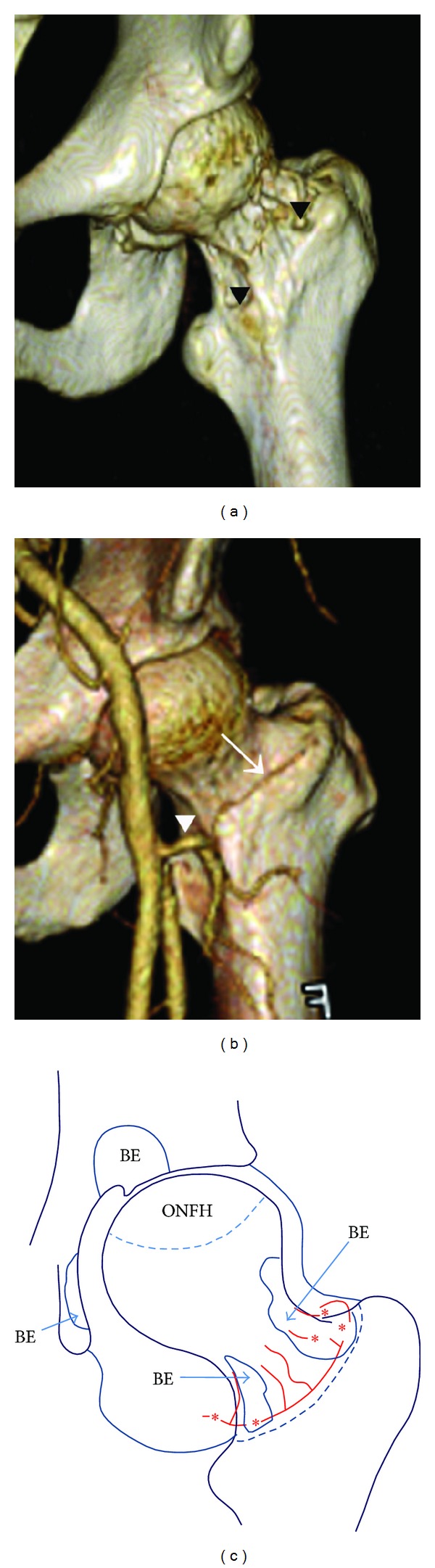
(a) Reconstructed three-dimensional computed tomography image shows bone erosions of the femoral head-neck junction (arrowhead). (b) Reconstructed three-dimensional computed tomography-angiography image of a normal individual. The lateral femoral circumflex artery (arrow) and base of medial femoral circumflex artery (arrowhead) are visible. (c) BE: bone erosion; ONFH: osteonecrosis of the femoral head. A schema of the BE area and ONFH. Asterisks show a break or compression of the lateral femoral circumflex artery, base of the medial femoral circumflex artery, and/or retinacular arteries. These arteries were considered to be injured by pigmented villonodular synovitis, creating multiple BE around the femoral head-neck junction.

Atraumatic ONFH progresses to the collapse of the femoral head in many patients and requires surgery to relieve hip pain. A systematic review in 2010 found that the causes of ONFH were corticosteroids (11%, 31/282), excessive alcohol consumption (16%), idiopathic disease (11%), systemic lupus erythematosus (21%), sickle cell disease (14%), renal failure and/or transplantation (17%), and human immunodeficiency virus (10%) [15]. According to our review of the English-language literature, no case reports of coincident ONFH and PVNS have been reported. We propose that the massive PVNS creating circular bone erosions around the femoral neck may have contributed to the pathogenesis of ONFH in the present case. Comparison of the three-dimensional (3D) CT image of the femoral neck lesion of this patient (Figure 3(a)) with a 3D CT angiographic image of a normal individual (Figure 3(b)) demonstrates the proximity of the bone erosion to the base of the medial and lateral femoral circumflex arteries. Schematic superimposition of the bone erosions and circumflex arteries (Figure 3(c)) suggests likely the compromise of the latter by the former.

In conclusion, we presented a detailed report of a case of ONFH that appeared to be secondary to PVNS. No recurrence of PVNS was detected for 3 years after the procedure.
